# Salidroside Mediated the Nrf2/GPX4 Pathway to Attenuates Ferroptosis in Parkinson’s Disease

**DOI:** 10.1007/s11064-024-04116-w

**Published:** 2024-02-29

**Authors:** Jun Shen, Shasha Chen, Xin Li, Lele Wu, Xue Mao, Jingjie Jiang, Dabu Zhu

**Affiliations:** 1 Department of General Medicine, Hangzhou Linping District First People’s Hospital, No. 369 Yingbin Road, Nanyuan Street, Linping District, Hangzhou, 311199 Zhejiang China; 2 Department of Medical Geriatrics, The Second Affiliated Hospital of Zhejiang University School of Medicine, Hangzhou, 310009 Zhejiang China; 3 Department of Pharmacy, Hangzhou Linping District First People’s Hospital, Hangzhou, 311199 Zhejiang China

**Keywords:** Salidroside, Nrf2/GPX4, Ferroptosis, Parkinson’s disease

## Abstract

Parkinson’s Disease (PD) is characterized by the loss of dopaminergic neurons, with ferroptosis playing a significant role. Salidroside (SAL) has shown neuroprotective potential, this study aims to explore its capacity to mitigate ferroptosis in PD, focusing on the modulation of the Nuclear Factor E2-Related Factor 2 (Nrf2)/ Glutathione Peroxidase 4 (GPX4) pathway. Male C57BL/6 mice were subjected to 1-methyl-4-phenyl-1,2,3,6-tetrahydropyridine (MPTP) to induce PD-like symptoms, followed by SAL and Nrf2 inhibitor administration. Then behavioral tests, immunohistochemical staining, transmission electron microscopy, and Western blot analysis were conducted to assess motor functions, pathological changes, ferroptosis, and related protein expressions. In vitro, SH-SY5Y cells were treated with erastin to induce ferroptosis to assess the protective effects of SAL. Additionally, A53T-α-synuclein (α-syn) was used to stimulate the PD model, SAL and a Nrf2 inhibitor (ML385) was utilized to elucidate the role of the Nrf2/GPX4 pathway in mitigating ferroptosis in PD. In vivo*,* SAL significantly improved motor functions and reduced the expression of α-syn, while increasing tyrosine hydroxylase (TH) expression of PD mice. Additionally, SAL treatment notably enhanced the levels of antioxidants and reduced MDA and iron content in the substantia nigra of PD mice. In vitro*,* SAL treatment increased the TH, GPX4, Nrf2 expression, and mitochondrial membrane potential whereas alleviated ferroptosis through the Nrf2/GPX4 pathway, as evidenced in erastin-induced and α-syn overexpressing SH-SY5Y cells. While these effects were reversed upon Nrf2 inhibition. SAL demonstrates significant potential in mitigating PD pathology and ferroptosis, positioning the Nrf2/GPX4 pathway as a promising therapeutic target. However, future studies should focus on the long-term effects of SAL, its pharmacokinetics, addressing the multifactorial nature of PD pathogenesis.

**Supplementary Information:**

The online version contains supplementary material available at 10.1007/s11064-024-04116-w.

## Introduction

Parkinson’s disease (PD) is a prevalent neurodegenerative disorder, affecting over 6 million individuals globally [1]. Its clinical manifestations encompass a broad spectrum of motor and non-motor symptoms, including resting tremor, bradykinesia, rigidity, cognitive impairments, and sleep disturbances [2]. Pathologically, PD is characterized by neural inclusions such as Lewy bodies and Lewy neurites, cell loss, and a reduction in dopaminergic neurons in the substantia nigra pars compacta, associated with systemic progressive iron accumulation [1]. Current PD drug therapies aim to enhance dopamine neurotransmission to alleviate symptoms [3]. The therapeutic arsenal includes dopamine precursors like L-DOPA, dopamine agonists, and dopamine metabolism inhibitors [4]. However, these drugs often lead to drug intolerance and insensitivity and cannot halt the pathophysiological progression of PD.

Ferroptosis has been implicated in various diseases, including cancer, neurodegenerative diseases, stroke, and PD [5]. In cells undergoing ferroptosis, ultrastructural changes in mitochondria are observed, including smaller mitochondria than normal, increased mitochondrial membrane density, reduced cristae, and outer membrane rupture [6]. Zeng et al. found that after administration of ferric ammonium citrate, Glutathione Peroxidase 4 (GPX4) significantly decreased and reactive oxygen species (ROS) significantly increased in PD cells, inducing ferroptosis leading to neuronal death [7]. In contrast, iron chelators can inhibit ferroptosis and protect neurons [8–10]. Additionally, research has found elevated iron levels in the substantia nigra pars compacta of PD patients, and reduced GPX4 expression in the substantia nigra pars compacta of deceased PD patients, which can lead to the ferroptosis of motor neurons, resulting in motor neuron degeneration and limb paralysis. Importantly, a double-blind randomized clinical trial has shown that deferoxamine treatment for PD is safe and effective [11]. Additionally, the α-synuclein (α-syn) protein, a major component of neuronal aggregates in PD, has been implicated in disease progression. Its interaction with iron ions and its role in ferroptosis through lipid metabolism pathways further emphasize the relevance of iron metabolism in PD [12–16].Nuclear Factor E2-Related Factor 2 (Nrf2) is a primary regulator of antioxidant responses and has recently been shown to prevent ferroptosis [17]. Under non-stress conditions, Nrf2 is continuously degraded via the ubiquitin–proteasome pathway in a keap1-dependent manner. In the presence of oxidative or electrophilic stress signals, the degradation of Nrf2 protein decreases, allowing it to accumulate in the nucleus, thereby upregulating the expression of many phase II detoxifying enzymes and antioxidant genes [18]. Under oxidative stress, Nrf2 translocates to the nucleus to induce the expression of endogenous antioxidant proteins responsible for preventing lipid oxidation. Nrf2 can control the expression of several iron metabolism proteins, GPX4, and other key proteins involved in glutathione biosynthesis [19, 20]. NF-kB, a key regulator of inflammatory responses, plays a significant role in neurodegenerative diseases. In PD, neuroinflammation is a critical factor contributing to disease progression. NF-kB’s involvement in inflammatory pathways suggests its potential impact on neuronal survival and ferroptosis. The activation of NF-kB in dopaminergic neurons can lead to the expression of pro-inflammatory cytokines, which may exacerbate neuronal damage. Therefore, understanding how SAL influences NF-kB activity could provide insights into its anti-inflammatory and neuroprotective effects in PD. In addition to Nrf2 and NF-kB, other transcription factors such as p53 and HIF-1 also contribute to the regulation of cellular responses in PD. For instance, p53 has been implicated in neuronal cell death, while HIF-1 plays a role in cellular responses to hypoxia, a condition that can exacerbate PD progression. Understanding how SAL affects these transcription factors could further elucidate its neuroprotective mechanisms.

*Rhodiola rosea L.* contains abundant bioactive components, including organic acids, flavonoids, tannic acid, and carbonic acid glycosides (such as salidroside (SAL), rosin, cinnamyl alcohol glycoside, etc.) [21]. Among them, SAL demonstrates favorable pharmacological effects, mainly including anti-aging, antioxidant, anti-inflammation, anti-cancer, and anti-depression [22]. Our previous research has found that SAL has a neuroprotective effect, it enhances autophagy through the mTOR/p70S6K signaling pathway, thereby reducing the expression of pathogenic β-amyloid protein and protecting SH-SY5Y cells [23]. Other studies have shown that it cannot only reduce the concentration of excitatory glutamate that causes cell apoptosis in rat hippocampal neurons but also downregulate the expression of the neurotoxic β-amyloid protein in SH-SY5Y neurons [24, 25]. Li et al. using the 1-methyl-4-phenyl-1,2,3,6-tetrahydropyridine (MPTP)-induced PC12 dopamine neuronal apoptosis model, found that SAL can inhibit ROS aggregation, thereby inhibiting the NO signaling pathway to alleviate MPTP damage to dopamine neurons [26]. Recently, Yang et al. found that activating the Nrf2/HO1 signaling pathway induced by Aβ1-42 in a mouse model of Alzheimer’s disease and glutamate-damaged HT22 cells can alleviate neuronal ferroptosis [27]. The above research indicates that SAL has a protective effect on neurons. However, the mechanisms underlying the neuroprotective effects of SAL are not fully understood and warrant further investigation.

Hence, this project explores SAL’s potential in upregulating Nrf2 and synthesizing GPX4 to mitigate neuronal ferroptosis. Utilizing SH-SY5Y cells and an A53T α-syn mutant PD model, we aim to assess SAL’s neuroprotection effects and its influence on Nrf2 expression. Concurrently, an MPTP-induced mouse PD model will help examine SAL’s protective role in vivo, observing its impact on damaged DA neurons and potential behavioral benefits. This research strives to elucidate SAL’s protective mechanisms on DA neurons, laying the groundwork for its future clinical applications in PD treatment.

## Materials and Methods

### Experimental Animals

All animal-related procedures were adhered to the Institutional Animal Care and Use Committee. Ethics approval was obtained from the Animal Experimentation Ethics Committee of Zhejiang Eyong Pharmaceutical R&D Center (Certificate No. SYXK (Zhe) 2021-0033). We utilized thirty-two 12-week-old male C57BL/6 mice according to the previous studies [28, 29] for consistent progression of PD symptoms, each weighing approximately 25 g, procured from SLAC, Shanghai, China (License No. SCXK (Hu) 2017-0005). These mice were subsequently accommodated in a Specific Pathogen-Free (SPF) environment at Zhejiang Eyong Pharmaceutical R&D Co., Ltd at a temperature of 24 °C, a relative humidity of 55%, and a 12-h light–dark cycle (Facility License: SYXK (Zhe) 2021-0003). They were provided with standard rodent pellet feed and unrestricted access to water and were exposed to a 12-h light cycle. Before drug administration, the mice underwent a week of acclimatization and were trained and screened for experimental animal behavior.

### PD Mouse Modeling

The PD mouse model was established by intraperitoneally injected MPTP (0388ES25, yeasen, Nanjing, China). The MPTP was dissolved in saline and was administered once daily for five consecutive days at the dosage of 30 mg/kg according to the previous study [30].

### Pharmacological Interventions and Groupings

**Fig. 1:**
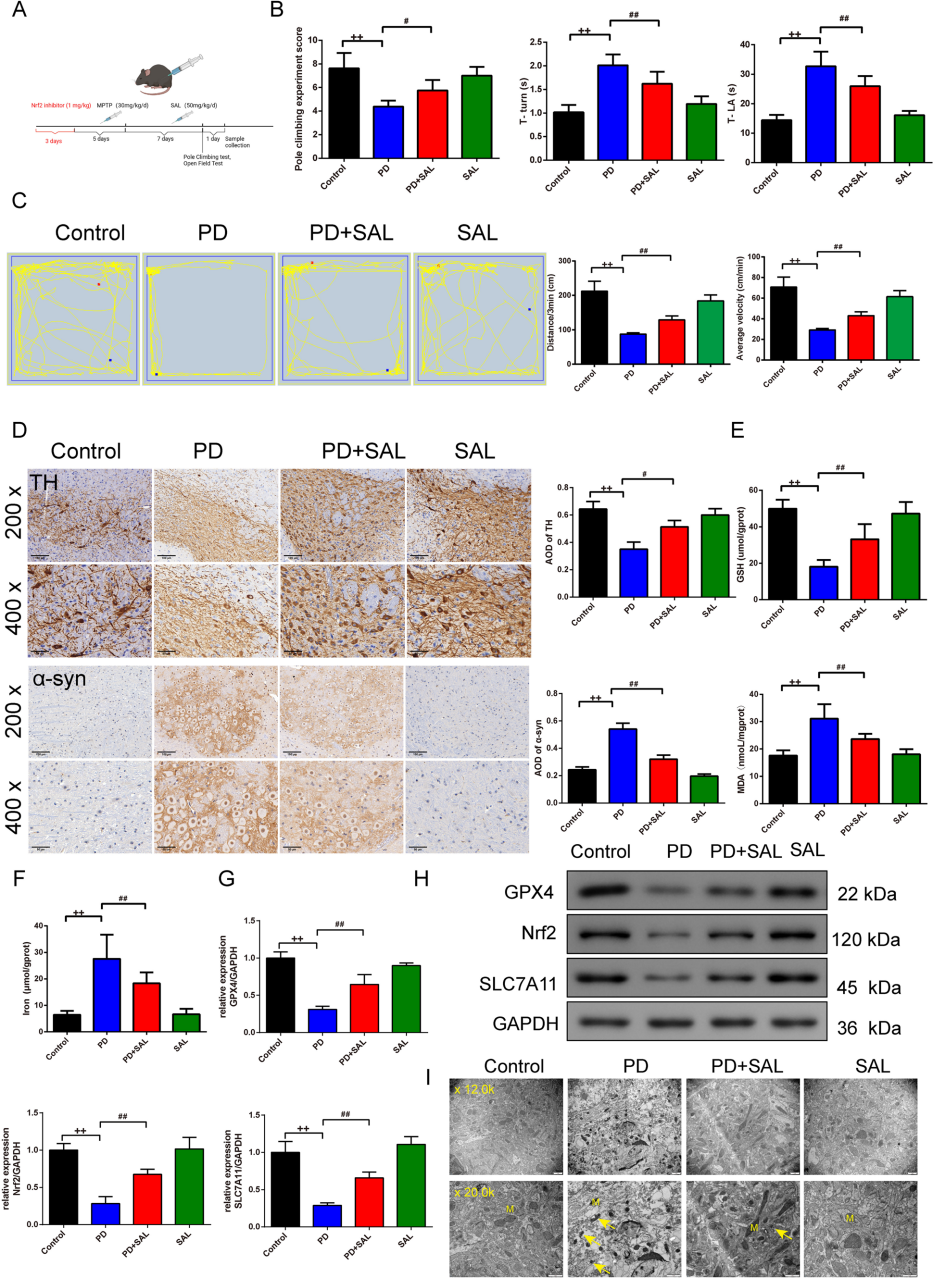
SAL protects dopaminergic neurons and inhibits ferroptosis of the substantia nigra in PD mice. **A** Flow chart of the experiment time. **B** The score of pole test comparison the groups, the time obtained for mice to turn completely downward (T-turn) and the time obtained for mice to climb to the floor (T-LA) were determined using the pole test, *n* = 8; **C** The track of mice 3 min total traveled distance (Distance/3 min) and average velocity (average velocity/3 min), *n* = 8; **D**The positive expression of the TH and α-syn in substantia nigra of the mice were stained by the IHC staining (magnification 200×, scale bar: 100 μm; magnification 400×, scale bar: 50 μm),* n* = 3; E: ELISA kits were applied to assess the level of the GSH and MDA in substantia nigra of the mice, *n* = 8; F: Iron level in substantia nigra of the mice was evaluated by the kit, *n* = 8; G-H: The protein expression of the GPX4, Nrf2, SLC7A11 was observed by the western blot, *n* = 3; I: TEM was used to observe the minor change in substantia nigra in mice, “M” stands for mitochondria (magnification 12.0 k× , scale bar: 1 μm; magnification 20.0 k×, scale bar: 1 μm); ^++^*P* < 0.01 vs. control group, ^#^*P* < 0.05 and ^##^*P* < 0.01 vs. PD group. *SAL* Salidroside, *PD* Parkinson’s disease, *TH* Tyrosine Hydroxylase, *IHC* Immunohistochemistry, *ELISA* Enzyme-Linked Immunosorbent Assay, *GSH* Glutathione, *MDA* Malondialdehyde, *GPX4* Glutathione Peroxidase 4, *Nrf2* Nuclear Factor E2-Related Factor 2, *SLC7A11* Solute Carrier Family 7 Member 11, *TEM* Transmission Electron Microscopy

SAL (S25475, yuanye, Shanghai), was administered intraperitoneally to mice at a dosage of 50 mg/kg/day over a continuous period of seven days [31]. The mice were divided into four main groups for the study (control, PD, PD+SAL, and SAL groups). The PD model group received an intraperitoneal injection of MPTP for five days to induce PD-like symptoms. The PD+SAL group, in addition to the MPTP injection, was also treated with SAL for seven consecutive days to explore the potential protective effects of SAL against PD. The SAL group served as a control, receiving only the SAL injection for seven days to observe its direct effects. For the control group in this study, the mice received intraperitoneal injections of a vehicle solution (saline) that matched the volume and frequency of the injections given to the other groups (PD, PD+SAL, SAL groups). Through this setup, the study aims to provide insights into the therapeutic potential of SAL in PD models. In addition, Nrf2 inhibitor (brusatol) (MedChemExpress) (1 mg/kg, i.p.) was injected 6 h before MPTP for three consecutive days. Brusatol was dissolved in 1% DMSO in a saline solution [32]. Flow chart of the whole experiment time was shown in Fig. 1A.

### Pole-Climbing Test

A wooden pole, 55 cm in length and 1 cm in diameter was vertically positioned. A 2 cm diameter foam ball was placed atop the pole, with both the ball and the pole wrapped in gauze to prevent the mice from slipping. The mice were placed head-down with their hind limbs on the ball. Scoring was as follows: 3 points for completion within 3 s, 2 points within 6 s, and 1 point for more than 6 s. Mice underwent two days of training before the actual test, with three trials daily. During the official test, each mouse was tested thrice, with a minute’s gap between each trial. The results were then averaged.

### Open Field Test

Mice were placed in a white square arena (45 × 45 × 60 cm) (ZS-KC, Zhongshi Dingsheng Biotechnology Co., LTD, Beijing). Their movements were recorded using a video camera positioned above the arena. The ANY-Maze animal behavior analysis system was employed to analyze the results, tracking the mice’s trajectory, distance, and average speed over a 3-min duration. The open field was sanitized with ethanol and air-dried between each test. All experiments were conducted blind.

### Sample Collection

24 h after the final administration and behavioral test, brain tissue from the nigral region was collected. Some of the brain tissue was used for paraffin section staining. The tissue was sliced into continuous coronal sections, each 5 μm thick. These slices were dried on a baking machine, arranged, and then stored in a 60 °C thermostat overnight. Another portion of the brain tissue from the substantia nigra was preserved at − 80 °C for upcoming molecular experiments.

### Cell Cultivation and Grouping

SH-SY5Y cells (iCell-h187, iCell Company) were meticulously cultured under DMEM/F12 medium enriched with 10% fetal bovine serum and 10 U/mL of penicillin–streptomycin at a stable 37 °C, with a 5% CO_2_. The cells were systematically categorized into distinct experimental groups. The erastin group was subjected to a 50 μM concentration of erastin (S80804-2 mg, MedMol) for 48 h. Concurrently, the SAL group was treated with 20 μM of SAL for an identical duration. Additionally, the erastin+Fer-1 group received a dual treatment, consisting of 1 μM Ferrostatin-1 (Fer-1, S81461, MedMol) alongside erastin, administered over a 48-h timeframe. To facilitate a comprehensive analysis, the cells were organized into specific treatment groups, namely: Control, erastin, SAL, erastin+SAL, and erastin+Fer-1.

### Plasmid Construction and Transfection

In the study, various plasmids were constructed and subsequently transfected into SH-SY5Y cells to analyze their effects. The control group was established using a mock plasmid, serving as an empty control. The WT-α-syn plasmid, containing the wild-type α-synuclein gene inserted into the pCDNA3.1(+) vector plasmid(NM_000345.4:226-648 Homo sapiens synuclein alpha (SNCA), transcript variant 1, mRNA, ATGGATGTATTCATGAAAGGACTTTCAAAGGCCAAGGAGGGAGTTGTGGCTGCTGCTGAGAAAACCAAACAGGGTGTGGCAGAAGCAGCAGGAAAGACAAAAGAGGGTGTTCTCTATGTAGGCTCCAAAACCAAGGAGGGAGTGGTGCATGGTGTGGCAACAGTGGCTGAGAAGACCAAAGAGCAAGTGACAAATGTTGGAGGAGCAGTGGTGACGGGTGTGACAGCAGTAGCCCAGAAGACAGTGGAGGGAGCAGGGAGCATTGCAGCAGCCACTGGCTTTGTCAAAAAGGACCAGTTGGGCAAGAATGAAGAAGGAGCCCCACAGGAAGGAATTCTGGAAGATATGCCTGTGGATCCTGACAATGAGGCTTATGAAATGCCTTCTGAGGAAGGGTATCAAGACTACGAACCTGAAGCCTAA). Additionally, the A53T-α-syn plasmid was created (SNCA MUT-A53T homo: ATGGATGTATTCATGAAAGGACTTTCAAAGGCCAAGGAGGGAGTTGTGGCTGCTGCTGAGAAAACCAAACAGGGTGTGGCAGAAGCAGCAGGAAAGACAAAAGAGGGTGTTCTCTATGTAGGCTCCAAAACCAAGGAGGGAGTGGTGCATGGTGTGaCAACAGTGGCTGAGAAGACCAAAGAGCAAGTGACAAATGTTGGAGGAGCAGTGGTGACGGGTGTGACAGCAGTAGCCCAGAAGACAGTGGAGGGAGCAGGGAGCATTGCAGCAGCCACTGGCT.

TTGTCAAAAAGGACCAGTTGGGCAAGAATGAAGAAGGAGCCCCACAGGAAGGAATTCTGGAAGATATGCCTGTGGATCCTGACAATGAGGCTTATGAAATGCCTTCTGAGGAAGGGTATCAAGACTACGAACCTGAAGCCTAA). Following the plasmid construction, the SH-SY5Y cells underwent transfection with these plasmids for a duration of 24 h, resulting in specific groupings: control, mock, WT-α-syn, and A53T-α-syn. Post transfection, the cells were subjected to a second round of treatment and grouping. They were treated with 20 μM of SAL for 48 h. In addition to this, a subset of SH-SY5Y cells was pre-treated with 10 μM of ML385, an Nrf2 inhibitor, for 2 h prior to SAL treatment. Following these treatments, the cells were collected for subsequent experimental analysis. The final groups for this stage were: Mock, WT-α-syn, A53T-α-syn, SAL, A53T-α-syn+SAL, and A53T-α-syn+SAL+ML385.

### Cell count Kit 8 (CCK8) Assay

SH-SY5Y cells were seeded in a 96-well plate and allowed to adhere overnight. After specific treatments, 10 μL of CCK8 solution (C0039, Beyotime, Shanghai) was added to each well. The plate was incubated at 37 °C for 2 h. Absorbance was measured at 450 nm using a microplate reader (CMaxPlus, MD, Shanghai).

### Immunohistochemical Staining

To prepare brain tissue sections, they were first deparaffinized in xylene and rehydrated through graded ethanol. Antigen retrieval was performed, followed by blocking to prevent non-specific binding. The sections were then incubated with primary antibodies (Tyrosine Hydroxylase (TH), ab137869, Abcam; α-syn, ab212184, Abcam) and secondary antibodies (IgG H&L (HRP), ab97080, Abcam), with PBS rinses in between. DAB substrate was applied for color development, monitored microscopically, and stopped with distilled water. Hematoxylin was used for counterstaining, followed by dehydration, clearing in xylene, and mounting. The stained sections were then ready for microscopic examination and image capture (Ts2-FC, Nikon, Japan).

### Western Blot

Proteins were extracted from SH-SY5Y cells and substantia nigra midbrain tissues prospectively and quantified with a BCA kit (pc0020, Solarbio). Equal amounts of protein were loaded onto SDS-PAGE gels and electrophoresed. Proteins were transferred to a PVDF membrane (10600023, GE Healthcare Life). The membrane was blocked with 5% milk. Primary antibodies (Table 1) incubation was done overnight at 4 °C. The membrane was washed and incubated with secondary antibodies (Table 1). Protein bands were visualized using chemiluminescence (610020-9Q, Clinx).

**Table 1:** Antibody information

Antibody	Company	Catalog number
GPX4 antibody	Affinity	DF6701
Nrf2 antibody	Affinity	BF8017
SLC7A11 antibody	Affinity	DF12509
α-syn antibody	Affinity	BF8041
Anti-mouse IgG, HRP-linked antibody	CST	7076
Anti-rabbit IgG, HRP-linked antibody	CST	7074
GAPDH antibody	proteintech	10494-1-AP

### Transmission Electron Microscopy (TEM)

Fix the brain tissue or SH-SY5Y cells in a solution of glutaraldehyde (R20515, yuanye, Shanghai), followed by post-fixation in osmium tetroxide to enhance contrast. After dehydration through a graded series of ethanol, infiltrate and embed the sample in an epoxy resin, ensuring it is well-penetrated. Once the resin is polymerized, use an ultramicrotome to cut ultra-thin sections of the sample, collecting them on copper grids. Stain the sections with uranyl acetate and lead citrate to increase electron density and contrast. Finally, examine the stained sections under a TEM (H-7650, Hitachi), adjust the focus and contrast as needed, and capture images for detailed cellular and subcellular structure analysis.

### Immunofluorescence Assay

The SH-SY5Y cells were fixed with paraformaldehyde, permeabilized with Triton X-100, and blocked with serum to prepare for immunofluorescence staining. Primary antibodies TH (ab137869, Abcam) and α-syn (ab138501, Abcam) were applied, followed by an overnight incubation at 4 °C. After washing off excess antibodies, a fluorescent secondary antibody, Goat Anti-Rabbit IgG H&L (Alexa Fluor 594) (ab150080, Abcam) was added. Post-wash, cell nuclei were stained with 4′,6-diamidino-2-phenylindole (DAPI). The cells were then imaged using a fluorescence microscope (Ts2-FC, Nikon, Japan).

### Enzyme-Linked Immunosorbent Assay (ELISA)

Malondialdehyde (MDA) and Glutathione (GSH) levels were measured using kits (A003-1, A006-2-1, Jiancheng, Nanjing), 4-hydroxynonenal (HNE), were measured utilizing kit (ELK8372, Elkbiotech, Wuhan). The procedure was conducted under the corresponding instruction, and reading absorbance at a designated wavelength (CMaxPlus, MD, Shanghai).

### Iron Content Detection

To measure the iron content in SH-SY5Y cells and brain tissues using the Solarbio Iron Content Assay Kit (BC5310, BC4350), start by thoroughly lysing the cells and tissues to ensure complete release of intracellular iron. Following cell lysis, centrifuge the samples to remove any debris, collecting the clear supernatant for analysis. Add the provided iron detection reagent to the supernatant according to the kit’s instructions, ensuring precise pipetting for accurate results. Incubate the mixture for a specified duration under the recommended conditions to allow for the complete reaction between iron ions and the detection reagent, resulting in a color change. After incubation, measure the absorbance of the samples using a spectrophotometer at the wavelength specified in the kit’s protocol (CMaxPlus, MD, Shanghai). Calculate iron content by comparing absorbance to a standard iron concentration curve.

### JC-1 Staining

The JC-1 mitochondrial membrane potential detection fluorescent probe (C2006, Beyotime, Shanghai) is used to detect changes in the mitochondrial membrane potential (MMP) of cells. JC-1 is dissolved in DMSO for packaging, with a concentration of 1 mg/mL, and stored at -20 ℃ in the dark. The SH-SY5Y cells and JC-1 were mixed evenly with PBS, and the final concentration of JC-1 was set to 5 μmol/L. After shaking and incubating at 37℃ for 15 min, the cells are washed with PBS and finally resuspended in 1 ml PBS. The red and green fluorescence is observed under a fluorescence microscope (LSM880, Zeiss). Normal mitochondria show orange-red fluorescence, indicating that the mitochondria are in a polarized state; under pathological conditions, green fluorescence is emitted. The intensity of the red and green fluorescence is analyzed using Image J software, and the mitochondrial membrane potential is represented by the red/green fluorescence intensity ratio.

### Statistical Treatment

Data analysis was conducted using SPSS 16.0 statistical software. For measurement data across multiple groups, we first assessed normality and homogeneity of variance. If these criteria were met, we proceeded with One-way ANOVA for variance analysis, followed by post-hoc pairwise comparisons using the Tukey test. In cases where data followed a normal distribution, but variances were unequal, we employed either Dunnett’s T3 test or the independent sample t-test. For data not adhering to a normal distribution, the Kruskal–Wallis H test was utilized. We set our significance level at α = 0.05. All results were expressed as mean ± standard deviation, with *P*-values less than 0.05 deemed to indicate statistical significance.

## Results

### SAL Protects Dopaminergic Neurons and Inhibits Ferroptosis of the Substantia Nigra Through Nrf2/GPX4 Pathway in PD Mice

The score of the pole-climbing test and the time of the T-turn, T-LA comparison of the groups in mice were recorded, we found that PD mice had lower scores (Fig. 1B, P = 0.000; F = 19.921, df = 3) as well as the lower T-turn(Fig. 1B, P = 0.001; F = 37.887, df = 3), T-LA (Fig. 1B, P = 0.001; F = 56.700, df = 3) than control mice. The treatment of SAL successfully enhanced the score, T-turn, and T-LA (Fig. 1B, P < 0.01).

In Fig. 1C, the open field test showed that the track of mice 3 min total traveled distance (Distance/3 min) (Fig. 1C, F = 77.188, df = 3, P = 0.000) and average velocity (average velocity/3 min) (Fig. 1C, F = 77.199, df = 3, P = 0.000)in PD mice were lower than control mice (Fig. 1C, P = 0.000) whereas the addition of SAL offset the effect that PD mice led to (Fig. 1C, P = 0.000).

The positive expression of the TH and α-syn in the substantia nigra of the mice were stained by the IHC staining (Fig. 1D). We observed that PD mice led to less expression of the TH (Fig. 1D, F = 19.821, df = 3, P = 0.000) whereas the more expression of the α-syn (Fig. 1D, F = 80.064, df = 3, P = 0.000) than control mice (Fig. 1D, P = 0.000). SAL adverse the effect that PD mice caused [Fig. 1D, P = 0.004 (TH), P = 0.000 (α-syn)].

Using ELISA kits and Western blot, we found that the level of the GSH (Fig. 1E, F = 46.647, df = 3, P = 0.000), GPX4 (Fig. 1G, H, F = 40.517, df = 3, P = 0.000), Nrf2(Fig. 1G, H, F = 31.606, df = 3, P = 0.000), and SLC7A11(Fig. 1G, H, F = 43.310, df = 3, P = 0.000) was reduced while MDA (Fig. 1E, F = 32.646, df = 3, P = 0.000)and iron content (Fig. 1F, F = 31.147, df = 3, P = 0.000)was enhanced in substantia nigra of the PD mice than control mice(P = 0.000), and SAL treatment offset the effects that PD mice caused [Fig. 1E–H, P = 0.002 (Nrf2, SLC7A11), P = 0.001 (GPX4, iron content) P = 0.000 (MDA,GSH)].

Under the TEM assay (Fig. 1I), the mitochondrial structure in the substantia nigra of the control group and SAL group was complete, and the shape was mostly round or oval. Compared with the control group, mitochondria in the substantia nigra of PD mice were irregular, the outer membrane was broken, wrinkled, and mitochondria were deeply stained. Compared with the model group, mitochondrial structure in the PD + SAL group was improved, and wrinkling was relieved. In addition, in Figure S1, with the addition of Nrf2 inhibitor, it reduced the score of the pole-climbing test (Figure S1 A, F = 17.216, df = 2, P = 0.000), the expression of GPX4 (Figure S1 C-D, F = 20.654, df = 2, P = 0.002), Nrf2 (Figure S1 C-D, F = 64.891, df = 2, P = 0.000), SLC7A11 (Figure S1 C-D, F = 124.328, df = 2, P = 0.000) tested by western blot, and it successfully enhanced T-turn (Figure S1 B, F = 48.113, df = 2, P = 0.000) and T-LA (Figure S1 B, F = 33.668, df = 2, P = 0.000) (Figure S1, P = 0.000, 0.001, 0.0002, 0.008, 0.010).

### SAL Protects Dopaminergic Neurons by Inhibiting Ferroptosis in SH-SY5Y Cells

**Fig. 2:**
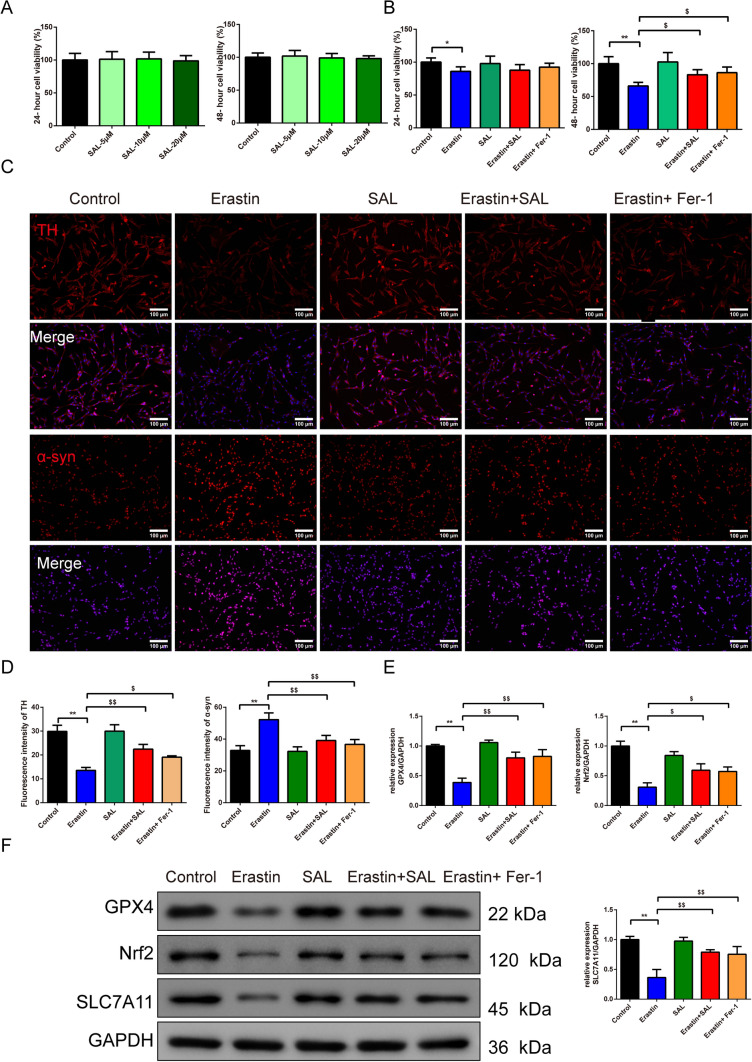
SAL protects dopaminergic neurons through inhibiting ferroptosis in SH-SY5Y cells. CCK8 was used to assess the cell viability of the SH-SY5Y cells under the different dosages (5, 10, 20 μm) of the SAL (**A**) and under the ferroptosis (**B**) in 24 h and 48 h, *n* = 6; **C**, **D** The TH and α-syn fluorescence intensity of SH-SY5Y cells inhibited by SAL and ferroptosis were detected by immunofluorescence (magnification 200×, scale bar: 100 μm),* n* = 3; **E**, **F** The protein expression of the GPX4, Nrf2, SLC7A11 of the SH-SY5Y cells was observed by the western blot, *n* = 3; ^*^P < 0.05 and ^**^P < 0.01 vs. control group, ^$^*P* < 0.05 and ^$$^*P* < 0.01 vs. erastin group. *SAL* Salidroside, *Fer-1* Ferrostatin-1, *CCK8* Cell Counting Kit-8, *TH* Tyrosine Hydroxylase, *GPX4* Glutathione Peroxidase 4, *Nrf2* Nuclear Factor E2-Related Factor 2, *SLC7A11* Solute Carrier Family 7 Member 11

In Fig. 2A, B, CCK8 was used to assess the cell viability of the SH-SY5Y cells under the various dosages (5, 10, 20 μM) of the SAL and under the ferroptosis in 24 and 48 h. The results demonstrated that the different dosages (5, 10, 20 μM) of SAL caused no side effects on the cell ability (Fig. 2A). Under the different treatments concerning erastin, SAL, and fer-1 in SH-SY5Y cells, the results showed that erastin induced lower cell viability than the control group in 24 h (Fig. 2B, F = 3.483, df = 4, P = 0.022) and 48 h (Fig. 2B, F = 13.281, df = 4, P = 0.000). Apart from that, after 48 h, erastin+SAL and erastin+Fer-1 groups had higher cell viability than the erastin group (Fig. 2B, P = 0.006, 0.001).

**Fig. 3:**
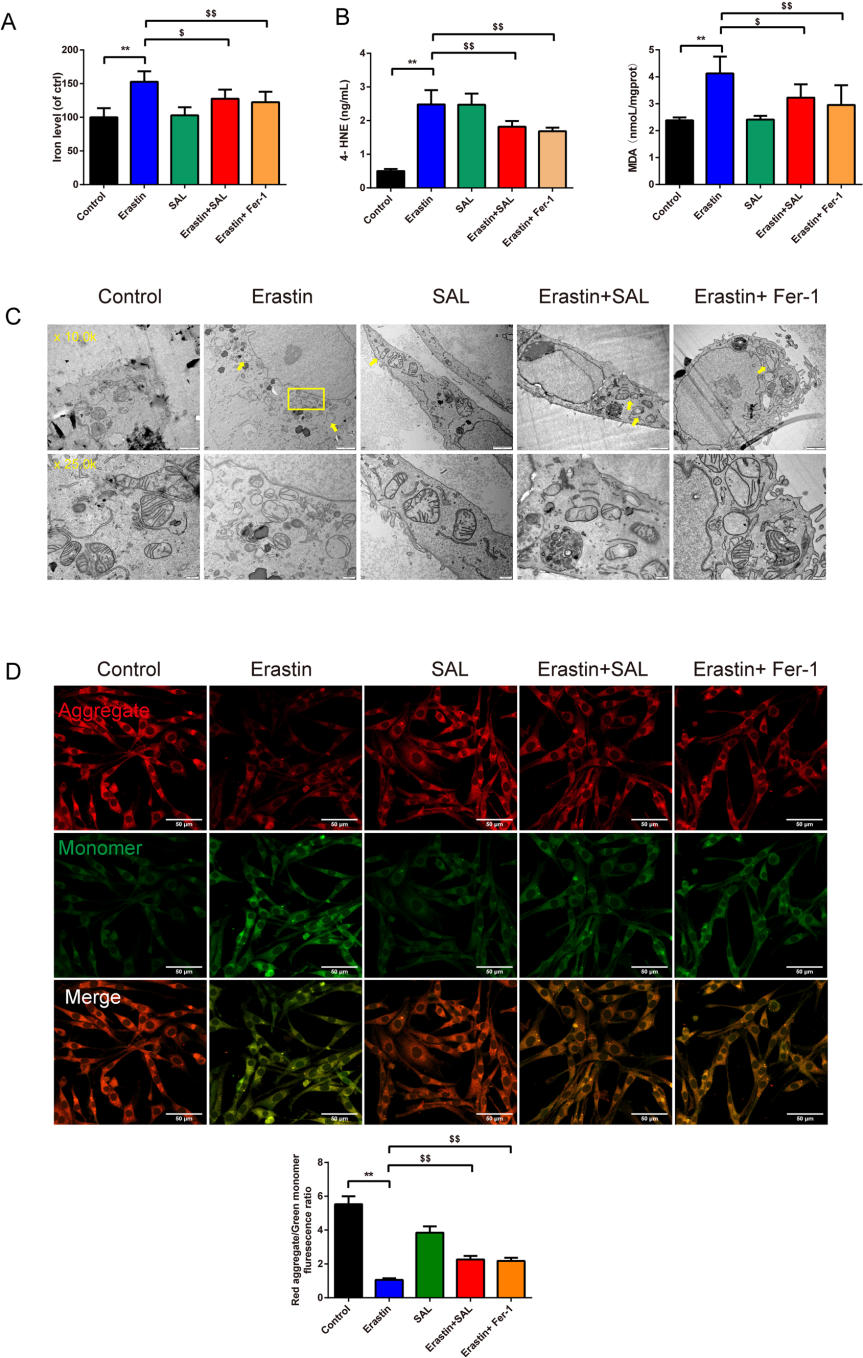
SAL protects dopaminergic neurons through inhibiting ferroptosis in SH-SY5Y cells. **A** Iron level in SH-SY5Y cells was evaluated by the kit, *n* = 8; **B** ELISA kits were applied to assess the level of the 4-HNE and MDA in SH-SY5Y cells, *n* = 8; **C** TEM was used to observe the minor change in SH-SY5Y cells, “M” stands for mitochondria (magnification 10.0 k×, scale bar 2 μm; magnification 25.0 k×, scale bar 500 nm); **D** The changes of MMP in SH-SY5Y cells were detected by JC-1 fluorescent probe (magnification 400×, 50 μm), *n* = 3. ^**^P < 0.01 vs. control group, ^$^*P* < 0.05 and ^$$^*P* < 0.01 vs. erastin group. *SAL* Salidroside, *Fer-1* Ferrostatin-1, *4-HNE* 4-Hydroxynonenal, *TH* Tyrosine Hydroxylase, *ELISA* Enzyme-Linked Immunosorbent Assay, *MDA* Malondialdehyde, *TEM* Transmission Electron Microscopy, *MMP* mitochondrial transmembrane potential

In Fig. 2C-F, the TH and α-syn fluorescence intensity in SH-SY5Y cells were detected by immunofluorescence staining and the protein expression of the GPX4, Nrf2, SLC7A11 in SH-SY5Y cells. Results revealed that the fluorescence intensity of TH (Fig. 2C, D, F = 38.091, df = 4, P = 0.000) and the relative protein expression of the GPX4 (Fig. 2E, F, F = 34.425, df = 4, P = 0.000), Nrf2(Fig. 2E, F, F = 32.083, df = 4, P = 0.000), and SLC7A11 (Fig. 2E, F, F = 22.470, df = 4, P = 0.000) in SH-SY5Y cells in the erastin group was lower than the control group (Fig. 2C–F, P = 0.000). Moreover, the erastin+SAL and erastin+Fer-1 groups led to a higher expression of those than the erastin group [Fig. 2C–F, P = 0.000(GPX4, SLC7A11), P = 0.002, 0.003(Nrf2)]. The expression of the α-syn (Fig. 2C, D, F = 18.010, df = 4, P = 0.000) showed the opposite impact. In Fig. 3A, B, the level of the iron, 4-HNE (Fig. 3B, F = 58.470, df = 4, P = 0.000), and MDA (Fig. 3B, F = 12.584, df = 4, P = 0.000) tested by ELISA kits was observed and they were expressed higher in the erastin group than control group (Fig. 3B, P = 0.000).whereas the lower expression in erastin + SAL and erastin + Fer-1 groups than erastin group [Fig. 3B, P = 0.005, 0.001 (iron); P = 0.004, 0.000 (MDA); P = 0.000 (4-HNE)].

In Fig. 3C, under TEM, the mitochondria of SH-SY5Y in the control and SAL groups were intact. Compared with the control, most of the mitochondrial ridges of SH-SY5Y in the erastin group were reduced or disappeared, and the outer membrane was ruptured. Compared with the erastin group, the erastin+SAL and erastin+fer-1 groups had improved mitochondrial ridge reduction or disappearance, outer membrane rupture, and volume reduction.

With the JC-1 staining (Fig. 3D), the MMP (Fig. 3D, F = 102.434, df = 4, P = 0.000) of SH-SY5Y cells in the erastin group was significantly decreased than control group (Fig. 3D, P = 0.000), while the MMP of SH-SY5Y cells in erastin+SAL group and erastin+Fer-1 groups was significantly increased than in erastin group (Fig. 3D, P = 0.001).

### SAL Alleviates Ferroptosis in SH-SY5Y Cells Through the Nrf2/GPX4 Pathway

**Fig. 4:**
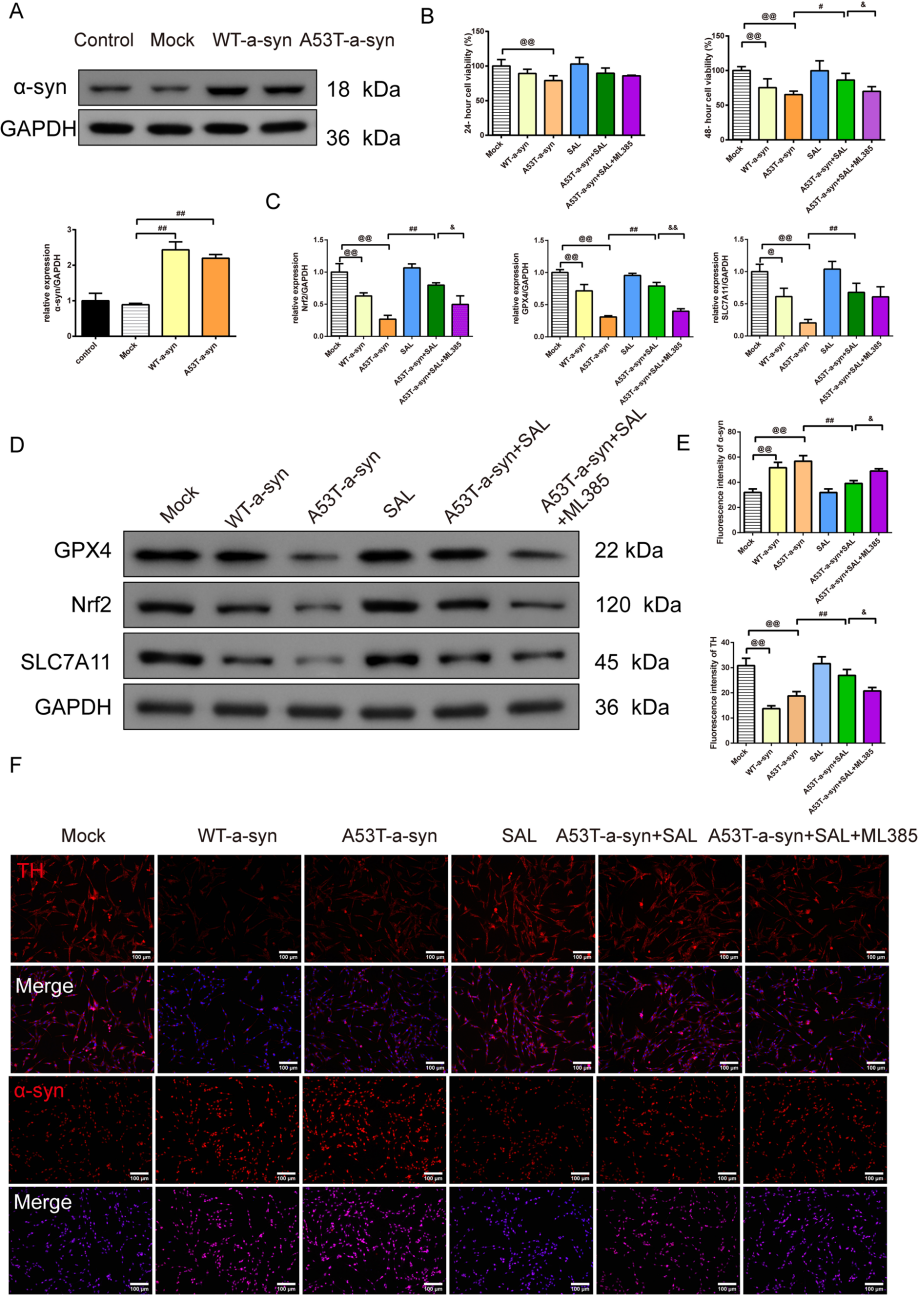
SAL alleviates ferroptosis in SH-SY5Y cells through the Nrf2/GPX4 pathway. **A** The protein expression of the α-syn in SH-SY5Y cells was observed by the western blot, *n* = 3; **B** CCK8 was used to assess the cell viability of the SH-SY5Y cells in 24 and 48 h with the interfere of the Nrf2 inhibition, *n* = 6, **C**, **D** the protein expression of the Nrf2, GPX4, SLC7A1 in SH-SY5Y cells was observed by the western blot, *n* = 3; **E**, **F** The TH and α-syn fluorescence intensity of SH-SY5Y cells with SAL and Nrf2 inhibition were detected by immunofluorescence (magnification 200×, scale bar: 100 μm),* n* = 3; ^@^*P* < 0.05 and ^@@^*P* < 0.01 vs. mock group, ^#^*P* < 0.05 and ^##^*P* < 0.01 vs. A53T-α-syn group, ^&^*P* < 0.05 and ^&&^*P* < 0.01 vs. A53T-α-syn + SAL group. *SAL* Salidroside, *CCK8* Cell Counting Kit-8, *TH* Tyrosine Hydroxylase, *GSH* Glutathione, *MDA* Malondialdehyde, *GPX4* Glutathione Peroxidase 4, *Nrf2* Nuclear Factor E2-Related Factor 2, *SLC7A11* Solute Carrier Family 7 Member 11

The protein expression of the α-syn (Fig. 4A, F = 71.656, df = 3, P = 0.000) in SH-SY5Y cells observed by Western blot showed that WT-α-syn and A53T-α-syn groups had higher expression of the α-syn than the mock group (Fig. 4A, P = 0.000).

In Fig. 4B, CCK8 was used to assess the cell viability of the SH-SY5Y cells at 24 and 48 h with the interference of the Nrf2 inhibition. The results confirmed that the A53T-α-syn group had lower cell viability in 24 h (Fig. 4B, F = 8.885, df = 5, P = 0.000) than the mock group (Fig. 4B, P = 0.000). In 48 h (Fig. 4B, F = 20.239, df = 5, P = 0.000), the WT-α-syn and A53T-α-syn groups had lower cell viability than the mock group (Fig. 4B, P = 0.000). The addition of the SAL enhanced the cell viability of than A53T-α-syn group (Fig. 4B, P = 0.001). The addition of ML385 offset the effect that SAL addition caused (Fig. 4B, P = 0.003).

**Fig. 5:**
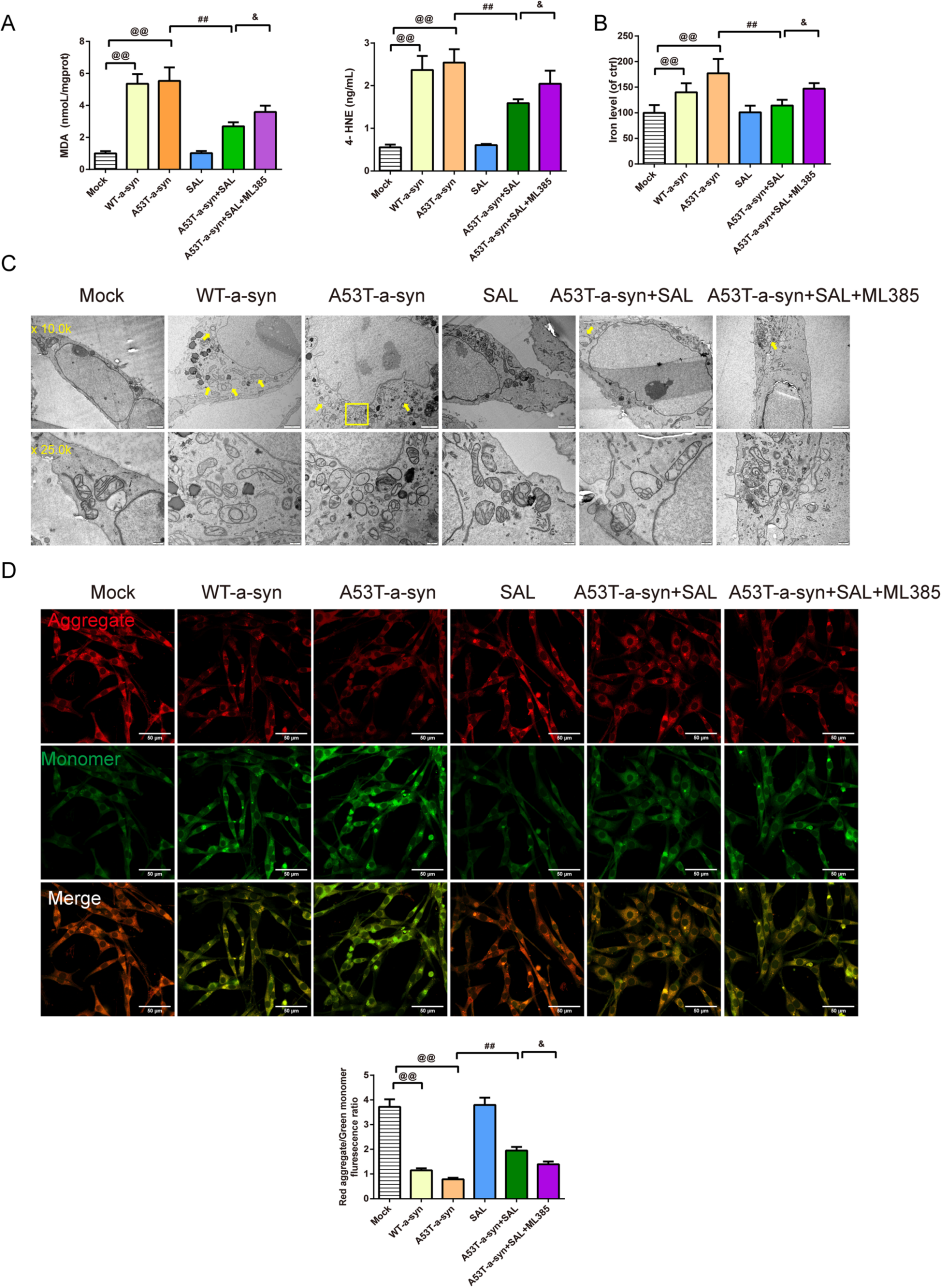
SAL alleviates ferroptosis in SH-SY5Y cells through the Nrf2/GPX4 pathway. **A** ELISA kits were applied to assess the level of the MDA and 4-HNE in SH-SY5Y cells, *n* = 8; **B** Iron level in SH-SY5Y cells evaluated by the kit, *n* = 8; **C** TEM was used to observe the minor change in SH-SY5Y cells (magnification 10.0 k×, scale bar: 2 μm; magnification 25.0 k×, scale bar: 500 nm); **D** the changes of MMP in SH-SY5Y cells were detected by JC-1 fluorescent probe (magnification 400 × , 50 μm), *n* = 3; ^@@^*P* < 0.01 vs. mock group, ^##^*P* < 0.01 vs. A53T-α-syn group, ^&^*P* < 0.05 vs. A53T-α-syn + SAL group. *SAL* Salidroside, *ELISA* Enzyme-Linked Immunosorbent Assay, *MDA* Malondialdehyde, *4-HNE* 4-Hydroxynonenal, *TEM* Transmission Electron Microscopy, *MMP* mitochondrial transmembrane potential

In Fig. 4C–F, the protein expression of the GPX4, Nrf2, SLC7A11, and the TH and α-syn fluorescence intensity in SH-SY5Y cells were detected by Western blot and immunofluorescence. The results revealed that the fluorescence intensity of TH (Fig. 4E, F, F = 33.267, df = 5, P = 0.000) and the relative protein expression of the GPX4 (Fig. 4C, D, F = 83.300, df = 5, P = 0.000), Nrf2 (Fig. 4C, D,F = 36.629, df = 5, P = 0.000), and SLC7A11 (Fig. 4C, D, F = 18.365, df = 5, P = 0.000) in SH-SY5Y cells in WT-α-syn and A53T-α-syn groups was lower than mock group (Fig. 4C, D, P = 0.000). Moreover, the addition of the SAL enhanced the expression of them than the A53T-α-syn group (Fig. 4C, D, P = 0.000, 0.001). The addition of ML385 offset the effect that A53T-α-syn + SAL caused (Fig. 4C, D, P = 0.000, 0.001). The expression of α-syn (Fig. 4E, F, F = 32.354, df = 5, P = 0.000) showed the opposite impact (P < 0.05 and P < 0.01). The trend of the MDA (Fig. 5A, F = 108.711, df = 5, P = 0.000), 4-HNE (Fig. 5A, F = 87.016, df = 5, P = 0.000), and iron content (Fig. 5B, F = 19.170, df = 5, P = 0.000) tested by the ELISA kits acted as α-syn expressed (Fig. 5A, B, P = 0.000, 0.002).

In Fig. 5C, under TEM, in SH-SY5Y cells of the Mock group, mitochondria were mostly intact. In comparison to the Mock group, in the WT-α-syn and A53T-α-syn groups, most mitochondria in SH-SY5Y dopaminergic neurons exhibited a reduction or loss of cristae and disruptions in the outer membrane. When comparing the A53T-α-syn group to the SAL group, mitochondria in SH-SY5Y dopaminergic neurons of the SAL group remained mostly intact, with only a slight reduction in cristae observed in the A53T-α-syn+SAL group. However, when comparing the A53T-α-syn+SAL group to the A53T-α-syn+SAL+ML385 group, there was an increased occurrence of reduced cristae in mitochondria.

With the JC-1 staining (Fig. 5D), the MMP (Fig. 5D, F = 136.018, df = 5, P = 0.000) of SH-SY5Y cells in WT-α-syn and A53T-α-syn groups was significantly decreased compared with the Mock group (Fig. 5D, P = 0.000); Compared with A53T-α-syn group, the MMP of SH-SY5Y cells in A53T-α-syn+SAL group was significantly increased (Fig. 5D, P = 0.000).Compared with the A53T-α-syn+SAL group, the MMP of SH-SY5Y cells in the A53T-α-syn+SAL+ML385 group was significantly increased (Fig. 5D, P = 0.005).

## Discussion

PD remains one of the most prevalent neurodegenerative disorders, characterized by the progressive loss of dopaminergic neurons in the substantia nigra [33]. The multifaceted nature of PD pathogenesis, encompassing protein aggregation, mitochondrial dysfunction, and oxidative stress, necessitates a comprehensive therapeutic approach [34, 35]. The present study delves into the potential neuroprotective effects of SAL in both in vivo and in vitro models of PD, shedding light on its promising therapeutic implications.

A critical observation from our study was the notable improvement in motor functions of PD mice post-SAL treatment. The pole test, a widely recognized behavioral assay for PD, revealed that SAL-treated mice exhibited enhanced motor coordination and agility [36], as evidenced by improved scores and reduced T-turn and T-LA times. One plausible explanation could be the restoration of dopaminergic transmission in the nigrostriatal pathway, a hallmark feature compromised in PD. The increased travel distances and velocities post-SAL treatment suggest a potential reversal of bradykinesia, a cardinal symptom of PD, which aligns with previous research emphasizing the importance of restoring dopaminergic transmission to alleviate PD-associated motor deficits as well as highlighting the motor-enhancing effects of various neuroprotective agents in PD models [30, 37]. In addition, TH, a rate-limiting enzyme in dopamine synthesis, typically shows decreased expression in PD [38], serving as a marker for the loss of dopaminergic neurons. In parallel, we observed that elevated levels of α-synuclein, a protein implicated in PD pathogenesis due to its tendency to form toxic aggregates [39], were significantly reduced following SAL treatment. This dual modulation of TH and α-synuclein by SAL highlights its potential as a targeted therapeutic strategy against the core pathological features of PD [40]. The mitochondrial protective effects of SAL, as evidenced by TEM, offer another dimension to its therapeutic potential [41]. The ability of SAL to preserve mitochondrial integrity, especially in the face of PD-induced damage, hints at its role in maintaining cellular energy homeostasis and preventing neuron loss. Additionally, SAL improved oxidative stress markers, with increased levels of GSH and decreased levels of MDA. The expression of GPX4, Nrf2, and SLC7A11 was also upregulated, indicating enhanced cellular antioxidant defenses. Consistently, it has been reported that Withania somnifera significantly ameliorate the typical markers of PD providing nigrostriatal dopaminergic neuroprotection by regulating oxidative stress and apoptosis mechanisms [42]. Previous research has linked Nrf2 activation to neuroprotection in various neurodegenerative disorders [43], suggesting that SAL’s ability to modulate Nrf2 could open new avenues for PD therapy.

SAL’s neuroprotective effects extend to dopaminergic neurons in SH-SY5Y cells [44], where it inhibits ferroptosis. When primary neurons were treated with erastin, there was a significant increase in ROS levels, a decrease in the expression of GPX4, and an increase in lipid peroxidation, all of which are hallmark features of ferroptosis [45]. Interestingly, the combined treatment of erastin and SAL showed a protective effect, indicating that SAL may counteract the ferroptotic processes induced by erastin. This is further supported by the comparison with the erastin+Fer-1 group, where Fer-1, a known inhibitor of ferroptosis, was used as a positive control for protection against erastin-induced cell death [46]. Our findings indicate that SAL not only protects SH-SY5Y cells from ferroptosis but also enhances their viability, especially in the presence of erastin. In another study, deferoxamine, an iron chelator, was shown to prevent ferroptosis and protect primary neurons from erastin-induced ferroptosis [47]. By drawing parallels between the protective effects of SAL in SH-SY5Y cells and the protective effects of deferoxamine in primary neurons, it becomes evident that both agents share a common mechanism of action in combating ferroptosis. This further solidifies the understanding that iron plays a central role in ferroptosis, and interventions aimed at modulating iron levels, or its associated metabolic processes can serve as effective strategies to mitigate ferroptotic cell death, particularly in the context of neurodegenerative diseases where dopaminergic neurons are at risk.

Apart from Wnt/β-catenin signaling for the treatment of neurodegenerative diseases [48], the Nrf2/GPX4 pathway plays a crucial role in cellular defenses against oxidative stress and ferroptosis. SAL’s ability to upregulate this pathway was evident in A53T-α-syn-SH-SY5Y cells, where it alleviated ferroptosis and promoted cell survival. Related, the research suggests that Morningside can activate the Nrf2/ARE signaling pathway to increase antioxidant capacity, thereby inhibiting abnormal lipid metabolism and protecting dopaminergic neurons in PD from ferroptosis [29]. Additionally, Sun observed a protective effect against 6-OHDA-induced dopaminergic neuronal ferroptosis in zebrafish and SH-SY5Y cell models through the upregulation of the Nrf2 signaling pathway [49]. Indeed, the Nrf2 signaling pathway has the potential to mitigate cellular oxidative damage, subsequently preventing ferroptosis in dopaminergic neurons affected by PD. In addition, with the Nrf2 inhibition (ML385), the protective effect of the SAL was the opposite. The study reported that the administration of Nrf2 activator dimethyl fumarate (DMF) led to a decrease in ROS levels, enhancing mitochondrial function and dendritic branching. Conversely, blocking Nrf2 with ML385 worsened these aspects [50]. Altering Nrf2 activity profoundly influenced mitochondrial health, oxidative stress response, and synaptic adaptability in A53TSyn neurons. These findings indicate that Nrf2 holds potential as a therapeutic target in PD.

In conclusion, our study provides a comprehensive analysis of the neuroprotective effects of SAL in PD. From behavioral improvements to molecular modulations, SAL emerges as a promising candidate for PD therapy. While our findings are robust, future studies should focus on the long-term effects of SAL, its pharmacokinetics, and potential side effects. Additionally, exploring its synergistic effects with existing PD medications could pave the way for a multi-targeted therapeutic approach, addressing the multifactorial nature of PD pathogenesis.

### Supplementary Information

Below is the link to the electronic supplementary material.

Supplementary file1 (TIF 3773 KB)—Fig. S1 SAL alleviates ferroptosis of the substantia nigra through the Nrf2/GPX4 pathway in PD mice. **A**, **B** The score of pole test comparison the groups, the time obtained for mice to turn completely downward (T-turn) and the time obtained for mice to climb to the floor (T-LA) were determined using the pole test, *n* = 8; **C** The protein expression of the GPX4, Nrf2, SLC7A11 was observed by the western blot, *n* = 3; ^##^P < 0.01 vs. PD group, ^$^P < 0.05, ^$$^P < 0.01 vs. PD+SAL group. *SAL* Salidroside, *PD* Parkinson’s disease, *GPX4* Glutathione Peroxidase 4, *Nrf2* Nuclear Factor E2-Related Factor 2, *SLC7A11* Solute Carrier Family 7 Member 11

## Data Availability

The datasets generated during and/or analyzed during the current study are available from the corresponding author upon reasonable request.

## References

[1] Tolosa E, Garrido A, Scholz SW, Poewe W (2021). Challenges in the diagnosis of Parkinson’s disease. The Lancet Neurology.

[2] Hayes MT (2019). Parkinson’s disease and Parkinsonism. Am J Med.

[3] Reichmann H (2023). Real-world considerations regarding the use of the combination of levodopa, carbidopa, and entacapone (Stalevo(®) ) in Parkinson’s disease. Eur J Neurol.

[4] Abe Y, Yagishita S, Sano H, Sugiura Y, Dantsuji M, Suzuki T, Mochizuki A, Yoshimaru D, Hata J, Matsumoto M, Taira S, Takeuchi H, Okano H, Ohno N, Suematsu M, Inoue T, Nambu A, Watanabe M, Tanaka KF (2023). Shared GABA transmission pathology in dopamine agonist- and antagonist-induced dyskinesia. Cell Rep Med.

[5] Li J, Cao F, Yin HL, Huang ZJ, Lin ZT, Mao N, Sun B, Wang G (2020). Ferroptosis: past, present and future. Cell Death Dis.

[6] Dixon SJ, Lemberg KM, Lamprecht MR, Skouta R, Zaitsev EM, Gleason CE, Patel DN, Bauer AJ, Cantley AM, Yang WS, Morrison B, Stockwell BR (2012). Ferroptosis: an iron-dependent form of nonapoptotic cell death. Cell.

[7] Zeng X, An H, Yu F, Wang K, Zheng L, Zhou W, Bao Y, Yang J, Shen N, Huang D (2021). Benefits of Iron Chelators in the treatment of Parkinson’s disease. Neurochem Res.

[8] Bai L, Yan F, Deng R, Gu R, Zhang X, Bai J (2021). Thioredoxin-1 rescues MPP(+)/MPTP-induced Ferroptosis by increasing glutathione peroxidase 4. Mol Neurobiol.

[9] Sun WY, Tyurin VA, Mikulska-Ruminska K, Shrivastava IH, Anthonymuthu TS, Zhai YJ, Pan MH, Gong HB, Lu DH, Sun J, Duan WJ, Korolev S, Abramov AY, Angelova PR, Miller I, Beharier O, Mao GW, Dar HH, Kapralov AA, Amoscato AA, Hastings TG, Greenamyre TJ, Chu CT, Sadovsky Y, Bahar I, Bayir H, Tyurina YY, He RR, Kagan VE (2021). Phospholipase iPLA2beta averts ferroptosis by eliminating a redox lipid death signal. Nat Chem Biol.

[10] Zuo Y, Xie J, Li X, Li Y, Thirupathi A, Zhang J, Yu P, Gao G, Chang Y, Shi Z (2021). Ferritinophagy-mediated ferroptosis involved in paraquat-induced neurotoxicity of dopaminergic neurons: implication for neurotoxicity in PD. Oxid Med Cell Longev.

[11] Devos D, Moreau C, Devedjian JC, Kluza J, Petrault M, Laloux C, Jonneaux A, Ryckewaert G, Garcon G, Rouaix N, Duhamel A, Jissendi P, Dujardin K, Auger F, Ravasi L, Hopes L, Grolez G, Firdaus W, Sablonniere B, Strubi-Vuillaume I, Zahr N, Destee A, Corvol JC, Poltl D, Leist M, Rose C, Defebvre L, Marchetti P, Cabantchik ZI, Bordet R (2014). Targeting chelatable iron as a therapeutic modality in Parkinson’s disease. Antioxid Redox Signal.

[12] Li HY, Liu DS, Zhang YB, Rong H, Zhang XJ (2023). The interaction between alpha-synuclein and mitochondrial dysfunction in Parkinson’s disease. Biophys Chem.

[13] Peng Y, Wang C, Xu HH, Liu YN, Zhou F (2010). Binding of alpha-synuclein with Fe(III) and with Fe(II) and biological implications of the resultant complexes. J Inorg Biochem.

[14] Carboni E, Tatenhorst L, Tonges L, Barski E, Dambeck V, Bahr M, Lingor P (2017). Deferiprone rescues behavioral deficits induced by mild iron exposure in a mouse model of alpha-synuclein aggregation. Neuromolecular Med.

[15] Fecchio C, Palazzi L, de Laureto PP (2018). Alpha-Synuclein and polyunsaturated fatty acids: molecular basis of the interaction and implication in neurodegeneration. Molecules.

[16] Ugalde CL, Lawson VA, Finkelstein DI, Hill AF (2019). The role of lipids in alpha-synuclein misfolding and neurotoxicity. J Biol Chem.

[17] Liu N, Lin X, Huang C (2020). Activation of the reverse transsulfuration pathway through NRF2/CBS confers erastin-induced ferroptosis resistance. Br J Cancer.

[18] Aramouni K, Assaf R, Shaito A, Fardoun M, Al-Asmakh M, Sahebkar A, Eid AH (2023). Biochemical and cellular basis of oxidative stress: implications for disease onset. J Cell Physiol.

[19] Kerins MJ, Ooi A (2018). The roles of NRF2 in modulating cellular iron homeostasis. Antioxid Redox Signal.

[20] Song X, Long D (2020). Nrf2 and ferroptosis: a new research direction for neurodegenerative diseases. Front Neurosci.

[21] Lu Y, Deng B, Xu L, Liu H, Song Y, Lin F (2022). Effects of *Rhodiola rosea* supplementation on exercise and sport: a systematic review. Front Nutr.

[22] Magani SKJ, Mupparthi SD, Gollapalli BP, Shukla D, Tiwari AK, Gorantala J, Yarla NS, Tantravahi S (2020). Salidroside—can it be a multifunctional drug?. Curr Drug Metab.

[23] Chen S, Cai F, Wang J, Yang Z, Gu C, Wang G, Mao G, Yan J (2019). Salidroside protects SHSY5Y from pathogenic alphasynuclein by promoting cell autophagy via mediation of mTOR/p70S6K signaling. Mol Med Rep.

[24] Zhang L, Yu H, Zhao X, Lin X, Tan C, Cao G, Wang Z (2010). Neuroprotective effects of salidroside against beta-amyloid-induced oxidative stress in SH-SY5Y human neuroblastoma cells. Neurochem Int.

[25] Li QY, Wang HM, Wang ZQ, Ma JF, Ding JQ, Chen SD (2010). Salidroside attenuates hypoxia-induced abnormal processing of amyloid precursor protein by decreasing BACE1 expression in SH-SY5Y cells. Neurosci Lett.

[26] Li X, Ye X, Li X, Sun X, Liang Q, Tao L, Kang X, Chen J (2011). Salidroside protects against MPP(+)-induced apoptosis in PC12 cells by inhibiting the NO pathway. Brain Res.

[27] Yang S, Xie Z, Pei T, Zeng Y, Xiong Q, Wei H, Wang Y, Cheng W (2022). Salidroside attenuates neuronal ferroptosis by activating the Nrf2/HO1 signaling pathway in Abeta1-42-induced Alzheimer’s disease mice and glutamate-injured HT22 cells. Chin Med.

[28] Yue M, Wei J, Chen W, Hong D, Chen T, Fang X (2022). Neurotrophic role of the next-generation Probiotic strain L. lactis MG1363-pMG36e-GLP-1 on Parkinson’s disease via inhibiting ferroptosis. Nutrients.

[29] Li M, Zhang J, Jiang L, Wang W, Feng X, Liu M, Yang D (2023). Neuroprotective effects of morroniside from Cornus officinalis sieb. Et zucc against Parkinson’s disease via inhibiting oxidative stress and ferroptosis. BMC Complement Med Ther.

[30] Zhang X, Zhang Y, Li R, Zhu L, Fu B, Yan T (2020). Salidroside ameliorates Parkinson’s disease by inhibiting NLRP3-dependent pyroptosis. Aging.

[31] Li R, Wang S, Li T, Wu L, Fang Y, Feng Y, Zhang L, Chen J, Wang X (2019). Salidroside protects dopaminergic neurons by preserving complex I activity via DJ-1/Nrf2-mediated antioxidant pathway. Parkinson’s Dis..

[32] Park JE, Leem YH, Park JS, Kim SE, Kim HS (2023). Astrocytic Nrf2 Mediates the neuroprotective and anti-inflammatory effects of Nootkatone in an MPTP-induced Parkinson’s disease mouse model. Antioxidants (Basel, Switzerland).

[33] GBD 2016 Neurology Collaborators (2019). Global, regional, and national burden of neurological disorders, 1990–2016: a systematic analysis for the Global Burden of Disease Study 2016. Lancet Neurol.

[34] Basu S, Song M, Adams L, Jeong I, Je G, Guhathakurta S, Jiang J, Boparai N, Dai W, Cardozo-Pelaez F, Tatulian SA, Han KY, Elliott J, Baum J, McLean PJ, Dickson DW, Kim YS (2023). Transcriptional mutagenesis of α-synuclein caused by DNA oxidation in Parkinson’s disease pathogenesis. Acta Neuropathol.

[35] Yang K, Yan Y, Yu A, Zhang R, Zhang Y, Qiu Z, Li Z, Zhang Q, Wu S, Li F (2024). Mitophagy in neurodegenerative disease pathogenesis. Neural Regen Res.

[36] Ghafarimoghadam M, Mashayekh R, Gholami M, Fereydani P, Shelley-Tremblay J, Kandezi N, Sabouri E, Motaghinejad M (2022). A review of behavioral methods for the evaluation of cognitive performance in animal models: current techniques and links to human cognition. Physiol Behav.

[37] Rai SN, Singh P (2020). Advancement in the modelling and therapeutics of Parkinson’s disease. J Chem Neuroanat.

[38] Kasanga EA, Han Y, Shifflet MK, Navarrete W, McManus R, Parry C, Barahona A, Nejtek VA, Manfredsson FP, Kordower JH, Richardson JR, Salvatore MF (2023). Nigral-specific increase in ser31 phosphorylation compensates for tyrosine hydroxylase protein and nigrostriatal neuron loss: implications for delaying Parkinsonian signs. Exp Neurol.

[39] Lai JI, Porcu A, Romoli B, Keisler M, Manfredsson FP, Powell SB, Dulcis D (2023). Nicotine-mediated recruitment of GABAergic neurons to a dopaminergic phenotype attenuates motor deficits in an alpha-synuclein Parkinson’s Model. Int J Mol Sci.

[40] Yadav SK, Rai SN, Singh SP (2017). Mucuna pruriens reduces inducible nitric oxide synthase expression in Parkinsonian mice model. J Chem Neuroanat.

[41] Li Y, Chen W, Wang D (2023). Promotion of mitochondrial fragmentation suppresses the formation of mitochondrial spherical compartmentation in PINK1(B9)*Drosophila melanogaster*. Biochem Biophys Res Commun.

[42] Prakash J, Chouhan S, Yadav SK, Westfall S, Rai SN, Singh SP (2014). *Withania somnifera* alleviates Parkinsonian phenotypes by inhibiting apoptotic pathways in dopaminergic neurons. Neurochem Res.

[43] Zhou Q, Chen B, Xu Y, Wang Y, He Z, Cai X, Qin Y, Ye J, Yang Y, Shen J, Cao P (2024). Geniposide protects against neurotoxicity in mouse models of rotenone-induced Parkinson’s disease involving the mTOR and Nrf2 pathways. J Ethnopharmacol.

[44] Li T, Feng Y, Yang R, Wu L, Li R, Huang L, Yang Q, Chen J (2018). Salidroside promotes the pathological α-synuclein clearance through ubiquitin-proteasome system in SH-SY5Y cells. Front Pharmacol.

[45] Wang L, Liu C, Wang L, Tang B (2023). Astragaloside IV mitigates cerebral ischaemia-reperfusion injury via inhibition of P62/Keap1/Nrf2 pathway-mediated ferroptosis. Eur J Pharmacol.

[46] Li JJ, Dai WQ, Mo WH, Xu WQ, Li YY, Guo CY, Xu XF (2023). Fucoidan ameliorates ferroptosis in ischemia-reperfusion-induced liver Injury through Nrf2/HO-1/GPX4 activation. J Clin Transl Hepatol.

[47] Zhang Y, Fan BY, Pang YL, Shen WY, Wang X, Zhao CX, Li WX, Liu C, Kong XH, Ning GZ, Feng SQ, Yao X (2020). Neuroprotective effect of deferoxamine on erastininduced ferroptosis in primary cortical neurons. Neural Regen Res.

[48] Ramakrishna K, Nalla LV, Naresh D, Venkateswarlu K, Viswanadh MK, Nalluri BN, Chakravarthy G, Duguluri S, Singh P, Rai SN, Kumar A, Singh V, Singh SK (2023). WNT-β catenin signaling as a potential therapeutic target for neurodegenerative diseases: current status and future perspective. Diseases (Basel, Switzerland).

[49] Sun Y, He L, Wang T, Hua W, Qin H, Wang J, Wang L, Gu W, Li T, Li N, Liu X, Chen F, Tang L (2020). Activation of p62-Keap1-Nrf2 pathway protects 6-hydroxydopamine-induced ferroptosis in dopaminergic cells. Mol Neurobiol.

[50] Brandes MS, Zweig JA, Tang A, Gray NE (2021). NRF2 activation ameliorates oxidative stress and improves mitochondrial function and synaptic plasticity, and in A53T α-synuclein hippocampal neurons. Antioxidants (Basel, Switzerland).

