# Supplementary figures and images for: Salidroside Mediated the Nrf2/GPX4 Pathway to Attenuates Ferroptosis in Parkinson’s Disease
Source: Neurochem Res. 2024 Feb 29;49(5):1291–305. doi: 10.1007/s11064-024-04116-w (PMC10991011; doi:10.1007/s11064-024-04116-w)

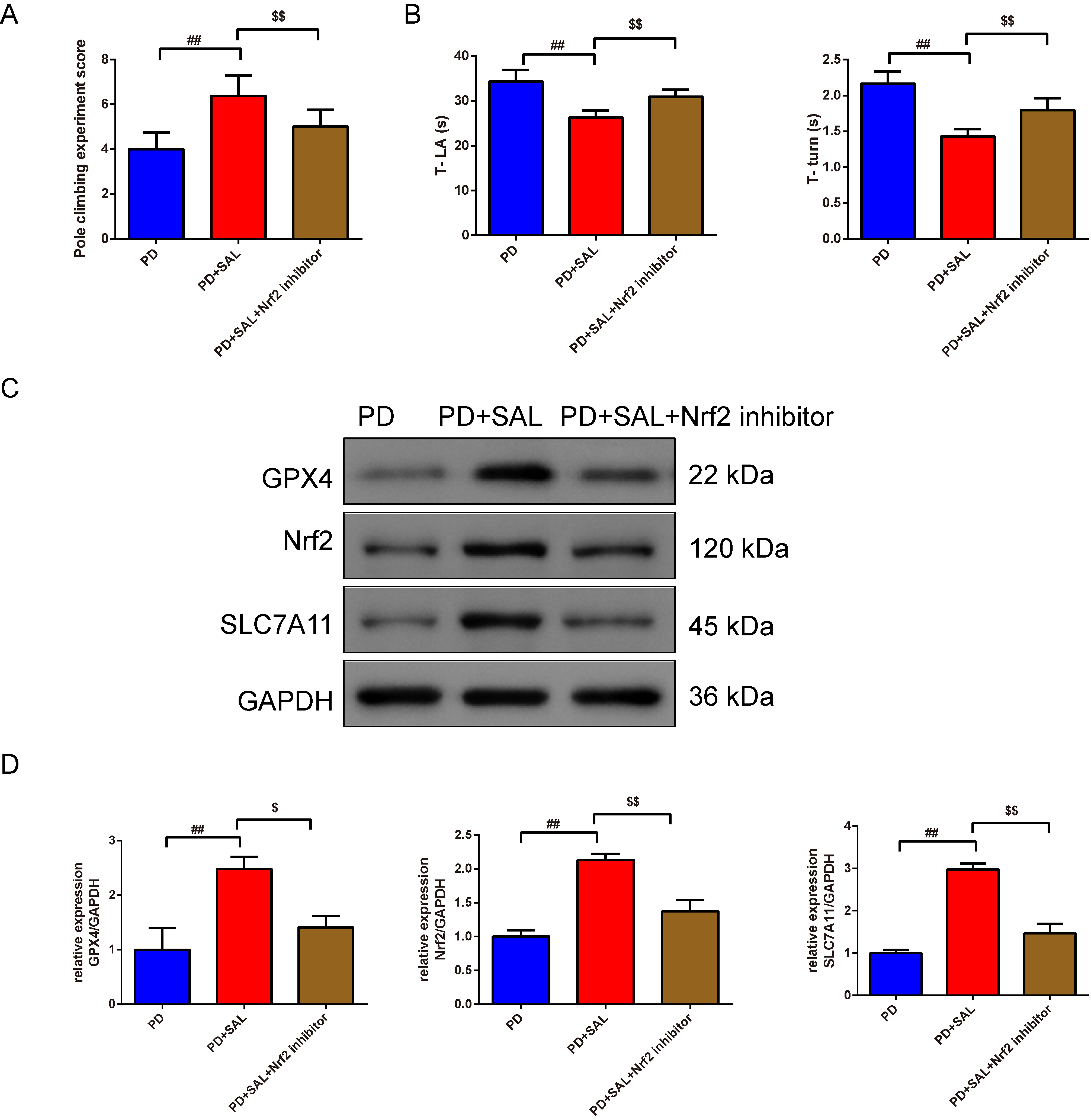

Supplement: Supplementary file 1 — Supplementary file1 (TIF 3773 KB)—Fig. S1 SAL alleviates ferroptosis of the substantia nigra through the Nrf2/GPX4 pathway in PD mice. A, B The score of pole test comparison the groups, the time obtained for mice to turn completely downward (T-turn) and the time obtained for mice to climb to the floor (T-LA) were determined using the pole test, n = 8; C The protein expression of the GPX4, Nrf2, SLC7A11 was observed by the western blot, n = 3; ##P < 0.01 vs. PD group, $P < 0.05, $$P < 0.01 vs. PD+SAL group. SAL Salidroside, PD Parkinson’s disease, GPX4 Glutathione Peroxidase 4, Nrf2 Nuclear Factor E2-Related Factor 2, SLC7A11 Solute Carrier Family 7 Member 11 [file 11064_2024_4116_MOESM1_ESM.tif]
